# Comparison between the Gametophyte and the Sporophyte Transcriptomes of the Endangered Fern *Vandenboschia speciosa*

**DOI:** 10.3390/genes14010166

**Published:** 2023-01-07

**Authors:** Rubén Martín-Blázquez, Mohammed Bakkali, Mercedes Ruiz-Estévez, Manuel A. Garrido-Ramos

**Affiliations:** 1 Department of Evolutionary Ecology, Estación Biológica de Doñana, Consejo Superior de Investigaciones Científicas (CSIC), Isla de la Cartuja, 41092 Sevilla, Spain; 2 Departamento de Genética, Universidad de Granada, 18071 Granada, Spain; 3 Corporate Research Materials Laboratory, 3M Center, Saint Paul, MN 55144, USA

**Keywords:** ferns, *Vandenboschia speciosa*, *de novo* transcriptome assembly, gene expression profile, functional annotation

## Abstract

Genomic resources are essential to understanding the evolution and functional biology of organisms. Nevertheless, generating genomic resources from endangered species may be challenging due to the scarcity of available specimens and sampling difficulties. In this study, we compare the transcriptomes of the sporophyte and the gametophyte of the endangered fern *Vandenboschia speciosa*. After Illumina sequencing and *de novo* transcriptome assembly of the gametophyte, annotation proved the existence of cross-species contamination in the gametophyte sample. Thus, we developed an *in silico* decontamination step for the gametophyte sequences. Once the quality check of the decontaminated reads passed, we produced a de novo assembly with the decontaminated gametophyte reads (with 43,139 contigs) and another combining the sporophyte and *in silico* decontaminated gametophyte reads (with 42,918 contigs). A comparison of the enriched GO terms from the top 1000 most expressed transcripts from both tissues showed that the gametophyte GO term set was enriched in sequences involved in development, response to stress, and plastid organization, while the sporophyte GO term set had a larger representation of more general metabolic functions. This study complements the available genomic resources on the life cycle of the endangered fern *Vandenboschia speciosa*.

## 1. Introduction

In the era of genomics, the amount of high throughput sequencing (HTS) resources for non-model organism has increased significantly in the last decade [1,2,3,4]. This trend eliminates possible artifacts caused by inferring non-model species based on model species and helps us understand the species-specific genomic or transcriptomic dynamics of non-model species. Differences exist even between con-generic species due to peculiarities during the processes of speciation, caused by adaptations to specific environmental conditions [5,6,7,8] or due to genome architecture dynamics [9,10]. Such differences might override characteristics present in a non-model species of interest that are not being captured by the life history of its phylogenetically closest model species. Thus, the genomic characterization of non-model organisms, including endemic/threatened species, is crucial to understanding possible differences between the genetic backgrounds of their more widespread and successful con-generics. As a study case, the endangered fern *Vandenboschia speciosa* illustrates the need to understand endemic species’ genomics and transcriptomics to better take decisions on their conservation.

*Vandenboschia speciosa* (Willd.) G. Kunkel (=*Trichomanes speciosum* Willd.), family Hymenophyllaceae, is considered one of the most vulnerable fern species in Europe. It is threatened by habitat destruction and excessive collection [11,12]. The ecological requirements of this species explain its current distribution, restricted to disjunctive populations in the European Atlantic stripe and the Macaronesian islands (Azores, Madeira, and the Canary islands), constituting a rare Macaronesian-European endemism. This species is the only representative in this area of the genus *Vandenboschia*, a genus of mainly tropical distribution. Its populations, composed of very few individuals, are found in places considered refuges for tertiary flora, suggesting their relic nature after the glacial cycles that occurred during the Tertiary. The species requires constantly humid and winter-warm conditions and is restricted to extreme low-light environments [13,14,15]. Both phases of the life cycle of *V. speciosa*, the sporophyte and the gametophyte, are perennial and can reproduce by vegetative propagation [14]. The sporophyte is rhizomatous and can propagate by fragmentation of its rhizome. Fronds are constituted by translucent leaves composed of a single layer of cells, thus having little control over water loss [13,14,15]. This species has adapted to grow in areas with low incidence of light and constant humidity. The gametophyte is epigeous and narrowly filamentous and can live in a wider range of habitats, including those that are darker and less humid. The adaptive responses allowing life in such conditions could be facilitated by morphological and physiological changes in the gametophyte [13,15]. Such sites can provide a microclimate and a stable environment for the long-term survival of independent gametophytes outside the sporophyte distribution range [14]. A mechanism consisting in the production of asexual propagules, called gemmae [15], has evolved in some populations of *V. speciosa* as well as in a few other species of the family Hymenophyllaceae to perpetuate the gametophyte by vegetative propagation without the intervention of the sporophyte [14,16,17,18].

Currently, there are three publicly available HTS projects for the genus *Vandenboschia*, two of which belong to *V. speciosa*. Generating transcriptomic resources for both the sporophyte and gametophyte of *V. speciosa* might shed light on the genetic regulation of the adaptive response of both life stages of this species [19,20,21,22] and could be of use for its conservation genetics. However, generating genomic resources from endangered species is challenging for some organisms due to sampling difficulties (i.e., low numbers of individuals, difficult access to their habitat, and a lack of high-quality nucleic acid extraction protocols). For instance, *V. speciosa* samples are rare and difficult to obtain, which makes it difficult to have replicates, especially when even one individual does not provide enough material for one replica—as in this case. In addition, gametophytes of *V. speciosa* are found in the ground in tight contact with ground and stream water, which makes RNA extraction conceivably contaminated by RNA from other (uni- and pluri-cellular) organisms, even when very high standards of careful and exhaustive isolation and cleaning of the filaments have been applied. In this study, we present a comparison between the *V. speciosa* gametophyte and sporophyte transcriptomes. The gametophyte transcriptome showed high inter-specific contamination levels due to the difficulty of collecting clean gametophyte tissue [22]. Thus, we performed *in silico* sequence decontamination steps to extract the most species-specific reads before the *de novo* transcriptome assembly of the gametophyte. We compared the *de novo* assemblies of the gametophyte transcriptome (before and after *in silico* decontamination) with the sporophyte transcriptome [19] and generate an additional *de novo* assembly using the decontaminated gametophyte and sporophyte reads.

## 2. Materials and Methods

### 2.1. Sample Collection and Sequencing

The details of sample collection and sequencing are detailed in [19]. In summary, five sporophytes and five gametophytes of *V. speciosa* were collected from Valdeinfierno (Cádiz, Spain). RNA was isolated from all ten specimens using the Spectrum™ Plant Total RNA Kit (Sigma, Madrid, Spain), and RNAs were pooled into a sporophyte RNA sample and a gametophyte RNA sample. The five samples were pooled for each life stage, resulting in two pooled samples: one for gametophytes and another for sporophytes. Both pooled sets of RNA were sequenced using Illumina HiSeq 2000 (Illumina Inc., San Diego, CA, USA) at Macrogen Inc. (Macrogen Inc., Seoul, Republic of Korea), generating paired-end reads. Illumina raw reads for sporophyte were already used in [19], and both sporophyte and gametophyte reads can be accessed at the Sequence Read Archive (SRA) of the NCBI under the accession numbers ERX2079928 (sporophyte) and ERX2079929 (gametophyte).

### 2.2. In Silico Decontamination of the Gametophyte Reads

*Vandenboschia speciosa* gametophyte reads were retrieved from a sample with non-specific material, which required extra steps of read curation before the analysis. The reads were mapped first to the *V. speciosa* sporophyte transcriptome using BWA (‘bwtsw’ reference indexing option and ‘sampe’ read alignment option) [23], keeping only reads with 99% identity or more for the downstream analyses. Reads mapped with less than 99% identity were aligned against the non-redundant (NR) NCBI sequence database (accessed on 12 March 2020) using DIAMOND and then analyzed with MEGAN6 to extract their taxonomical information [24]. The reads that matched the taxonomic tag Polypodiidae were kept for further analyses. We did not include “broader” taxonomical categories (i.e., Viridiplantae) nor mapped the remaining reads to other fern genomes to avoid including potentially cross-contaminated sequences from other plant species whose tissues might be present in the sample (pollen, spores, tissue remnants, etc). Both the raw reads and the *in silico* decontaminated reads were used to perform a *de novo* transcriptome assembly using Trinity [25].

### 2.3. V. speciosa De Novo Transcriptome Assembly

We used Trinity v2.13 [25] to perform *de novo* transcriptome assembly with the gametophyte raw reads alone, the gametophyte *in silico* decontaminated reads, and an additional *de novo* transcriptome assembly using both the sporophyte and gametophyte in silico decontaminated reads. To evaluate how the *in silico* decontamination step went for the gametophyte assembly, we used BLASTx [26,27] against UniProt (accessed on 16 May 2019) to align the contigs of the three unprocessed Trinity assemblies and compared the proportion of plant, animal, fungi, and protozoa from the 50 most represented species BLAST hits between the sporophyte, raw gametophyte, *in silico* decontaminated gametophyte, and combined assemblies. In addition, we compared the percentage of sequences with a positive BLAST hit against any sequence from *Arabidopsis thaliana* to show additional evidence for the *in silico* decontamination step.

The *in silico* decontaminated gametophyte and combined sporophyte and *in silico* decontaminated gametophyte reads were then analyzed. We calculated the sequencing depth of each assembled contig and estimated the expression in transcripts per million (TPMs) using Salmon [28]. We calculated N50 and ExN50 statistics of the transcriptomes using the Trinity script contig_ExN50_statistic.pl [25]. Then we removed contigs with TPM < 1 using the Trinity script filter_low_expr_transcripts.pl [25], mapped them to the UniProt sequence database using BLASTx, and used the BLAST results to calculate the BLAST result distribution per contig coverage using the Trinity script analyze_blastPlus_topHit_coverage.pl [25]. We used CD-HIT-EST [29] with c = 0.95 and n = 8 to remove redundant contigs and repeated the quality check steps described above to evaluate the assembly quality after CD-HIT-EST.

### 2.4. Assessment of Transcriptome Completion, Coding Sequence Presence, and Functional Annotation

We ran BUSCO analyses [30,31] in both transcriptomes, using the lineage databases Eukaryote, Viridiplantae, and Embryophyta from OrthoDB (www.orthodb.org; accessed on 27 of July, 2022), to assess the completeness of the assembly. We used TransDecoder [25] to predict coding domain sequences (CDSs) in the contigs. We compared the transcriptome statistics of both the gametophyte alone and the combination of the gametophyte and sporophyte with the already published sporophyte transcriptome [19]. Gene ontology (GO) term annotation was carried out using the GO term annotation from *A. thaliana* by running BLASTx with our combined gametophyte and sporophyte transcriptome against the *A. thaliana* protein set and then retrieving the GO terms (hosted in the Gene Ontology Consortium page, accessed on 28 June 2022) associated to each *A. thaliana* protein.

### 2.5. Transcriptome Expression Profile

We used BWA (‘bwtsw’ reference indexing option and ‘sampe’ read alignment option) to align the *in silico* decontaminated gametophyte and sporophyte reads to the combined gametophyte and sporophyte transcriptome. After editing the resulting files with samtools [32], we summarized the counts with htseq-count [33] (using the “interception non-empty” method). Normalized expression values were calculated using the DESeq2 [34] R package. The logarithm of fold change (logFC) was calculated by dividing each transcript’s gametophyte normalized counts by its sporophyte normalized counts, then taking the logarithm to base two. Lists of transcripts expressed in the gametophyte and the sporophyte were compared using Fisher’s exact test, and the overlap was illustrated in a Venn diagram generated with the online tool hosted at “http://www.interactivenn.net/ (accessed on 27 of July, 2022)”.

GO term enrichment analysis was performed through the Gene Ontology Resource website (“http://geneontology.org/ (accessed on 29 of June, 2022)”), using PANTHER v14 [35,36], selecting *A. thaliana* as the background dataset, applying the Fisher’s exact test, and using false discovery rate (FDR) corrected *p*-values [37]. We compared the 1000 most expressed transcripts in the gametophyte and the 1000 most expressed transcripts in the sporophyte through GO term enrichment analysis results. We used ReViGO [38] to remove redundant GO terms from the enriched GO term lists by selecting for a small-sized list of filtered GO terms and searching only in the *A. thaliana* protein database.

## 3. Results and Discussion

### 3.1. Cleaning up Cross-Contamination in the Gametophyte Reads

After mapping the gametophyte reads to the sporophyte transcriptome, a total of 24,733,606 read pairs (50.74%) of them mapped with a 99% identity or more. Regarding the paired reads that showed less than 99% identity, Diamond alignment matched 5.4 million of them (22.5%) to at least one target sequence from the NR database, but only 33.9 thousand reads matched to sequences from Polypodiidae. The total number of reads and total bases sequenced are shown in Table 1.

### 3.2. De Novo Assembly of the V. speciosa Transcriptome

**Figure 1:**
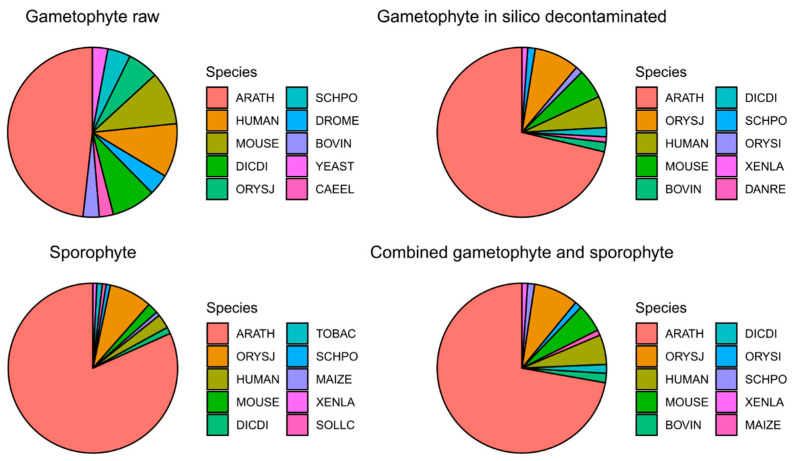
Species distribution of BLAST results from *V. speciosa* transcriptomes. The top 10 most represented species from BLASTx analysis against UniProt are shown for each transcriptome. ARATH: *Arabidopsis thaliana*. HUMAN: *Homo sapiens sapiens*. MOUSE: *Mus musculus*. DICDI: *Dictyostelium discoideum*. ORYSJ: *Oryza sativa* subsp. *japonica*. SCHPO: *Schizosaccharomyces pombe*. ORYSI: *Oryza sativa* subsp. *indica*. DROME: *Drosophila melanogaster*. BOVIN: *Bos taurus*. YEAST: *Saccharomyces cerevisiae*. CAEEL: *Caenorhabditis elegans*. XENLA: *Xenopus laevis*. DANRE: *Danio rerio*. TOBAC: *Nicotiana tabacum*. MAIZE: *Zea mays*. SOLLC: *Solanum lycopersicon*.

The de novo assembly generated 203,306 contigs for the raw gametophyte transcriptome, 44,455 contigs for the *in silico* decontaminated gametophyte transcriptome, and 88,383 contigs for the combined sporophyte and gametophyte transcriptome. The 50 most represented species in the BLAST hits from the raw gametophyte transcriptome showed 30% of plant species, while the *in silico* decontaminated gametophyte, combined sporophyte and gametophyte, and sporophyte assemblies showed, respectively, 60%, 60%, and 68% of plant species (Table 2). We did not expect a close to 100% plant result in this analysis since the UniProt database includes a selected high-quality annotated protein set, not necessarily including all the proteins from the plant genomes in the database (i.e., transcripts from *V. speciosa*, whose best hit is a non-plant protein due to a lack of homologous sequences in UniProt). Tracking down the percentage of BLAST hits assigned to *A. thaliana*, we found that only 30.29% of the BLAST hits in the raw gametophyte assembly belong to that species, while in the *in silico* decontaminated gametophyte, combined sporophyte and gametophyte, and sporophyte assemblies, the percentages are 55.88%, 55.82%, and 70.00% (Table 2). This trend is also shown in Figure 1, where the ten most represented species in the BLAST results for the transcriptomes assembled in this study show a higher proportion of *A. thaliana* BLAST hits when comparing the raw gametophyte transcriptome with the others. We discarded the raw gametophyte assembly due to its high species cross-contamination.

**Figure 2:**
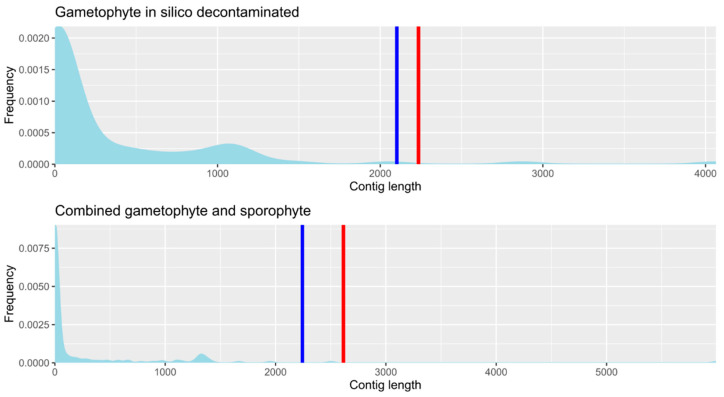
Contig length distribution from the gametophyte and the combined transcriptomes. The X axis shows the contig lengths. The Y axis shows the frequency of contigs (transformed to proportion) for each contig length value. The blue vertical line shows the N50 value, and the red vertical line shows the Ex90N50 value.

After filtering the contigs by sequencing depth and removing redundant contigs, the *in silico* decontaminated gametophyte and the combined sporophyte and gametophyte transcriptomes contained 43,139 and 42,918 contigs, respectively. Contig length distribution and the N50 and Ex90N50 values are shown for both transcriptomes in Figure 2. Details of transcriptome statistics from both transcriptomes, before and after contig filtering, as well as from the already published sporophyte transcriptome [19], are shown in Table 3. The N50 value was comparable to those of other plant de novo assembly transcriptome projects, including ferns [39,40,41,42,43,44,45]. The difference in contig number between transcriptomes can be attributed to (i) the number of *in silico* decontaminated gametophyte reads being half that of sporophyte reads, so a transcript with a TPM value close to but higher than one in the *in silico* decontaminated gametophyte transcriptome could have dropped its TPM value below one in the combined gametophyte and sporophyte transcriptome, thus being filtered after not reaching the expression threshold (TPM > 1) to be considered a valid transcript; and (ii) the gametophyte transcriptome has more fragmentation compared to the combined transcriptome, so sporophyte reads might have contributed to fill in the gaps of these gametophyte partial transcripts, thus reducing the total number of sequences. This last option is supported by the higher values of N50 and Ex90N50 in the combined transcriptome, which indicate higher contig lengths compared to de novo assemblies generated from individual tissues (Table 3). Overall, the net number of transcripts lost between *in silico* decontaminated gametophyte and combined sporophyte assemblies was lower than 0.4%. Supplemental Figure S1 shows both *in silico* decontaminated gametophyte and combined gametophyte and sporophyte transcriptome read coverage.

**Figure 3:**
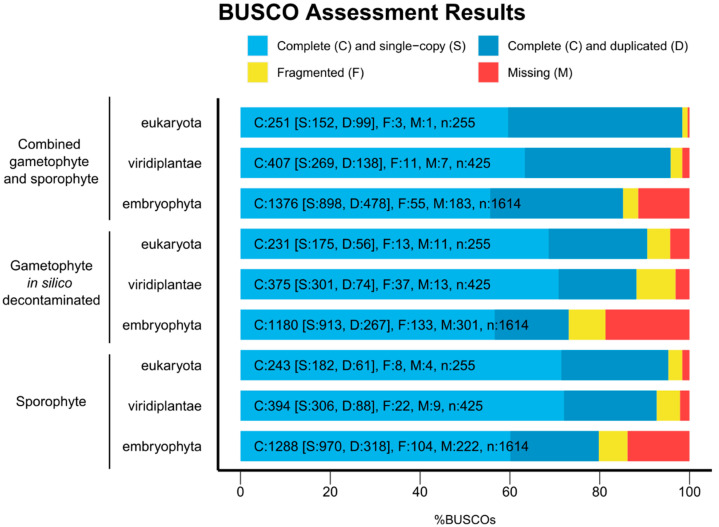
BUSCO assessment results. The X axis shows the percentage of BUSCOs found for each analysis. The Y axis shows the transcriptome (1st column) and BUSCO taxonomical level (2nd column). Each color-coded bar shows the number of complete (C, including the ones found as single copies, S, and multiple copies, D), fragmented (F), and missing (M) BUSCOs from the total analyzed (n).

The BUSCO analysis showed always less than 20% (from 18.65% to 0.39%) of missing BUSCOs for all the transcriptomes and for the BUSCO databases Eukaryota, Viridilantae and Embryophyta (Figure 3). The *in silico* decontaminated gametophyte transcriptome showed the lowest number of complete BUSCOs (90.58% Eukaryota, 88.24% Viridiplantae, 73.11% Embryophyta), whereas the combined sporophyte and gametophyte transcriptome showed a higher number of completed BUSCOs (98.43% Eukaryota, 95.77% Viridiplantae, 85.25% Embryophyta), slightly surpassing the numbers of the sporophyte transcriptome (95.29% Eukaryota, 92.71% Viridiplantae, 79.80% Embryophyta), supporting that complete general species transcriptomes should include sequencing from multiple tissues [31,46]. The proportion of duplicated complete BUSCOs in the combined gametophyte and sporophyte transcriptome increased compared to both single tissue transcriptomes (33.91–39.44% complete duplicated BUSCOs in the combined transcriptome, 19.73–24.24% in the *in silico* decontaminated gametophyte transcriptome, and 22.34–25.10% in the sporophyte transcriptome). Other transcriptome assemblies from fern species recovered between 53% and 71% of complete Embryophyta BUSCOs, even when including RNA-seq libraries from several tissues [43,44,47]. This new version of the *V. speciosa* transcriptome completes the transcriptome of the sporophyte [19], increasing the total contig count to 6,488 sequences and increasing the percentage of Eukaryote, Viridiplantae, and Embryophyta BUSCOs to 3.14%, 3.06%, and 5.45%, respectively. Taken together, the BUSCO results and transcript contiguity measures (Table 3) indicate that we have an acceptable transcriptome assembly.

After a search for coding sequences (CDSs) with TransDecoder, the *in silico* decontaminated gametophyte showed 24,343 CDSs, 14,968 of them complete (61.49%), and the rest being truncated at their 5′ end, 3′ end, or both. The combined gametophyte and sporophyte transcriptome showed 32,726 CDSs, 23,987 of which were completed (73.30%).

BLAST analysis using UniProt as a reference database retrieved 34,405 positive hits for the *in silico* decontaminated gametophyte transcriptome and 35,712 for the combined gametophyte and sporophyte transcriptome. Table 4 summarizes the number of proteins retrieved before and after filtering the transcriptomes in function of the percentage of identity covered between the query (*V. speciosa* transcripts) and the target (UniProt database) sequences. As expected, the combined transcriptome surpassed the gametophyte transcriptome in number of assigned proteins for all intervals; however, the combined tissue assembly showed fewer assigned proteins. As mentioned above, this is due to the presence of transcripts with low coverage that passed filtering based on TPM in the gametophyte transcriptome but were purged in the combined tissue transcriptome due to their TPM value readjustment being lower than one. We retrieved 5924 GO terms using the *A. thaliana* genome protein set (27,136 *V. speciosa* transcripts with 10,961 BLAST hits from the combined gametophyte and sporophyte transcriptome).

### 3.3. Differences in Transcript Expression between Tissues

**Figure 4:**
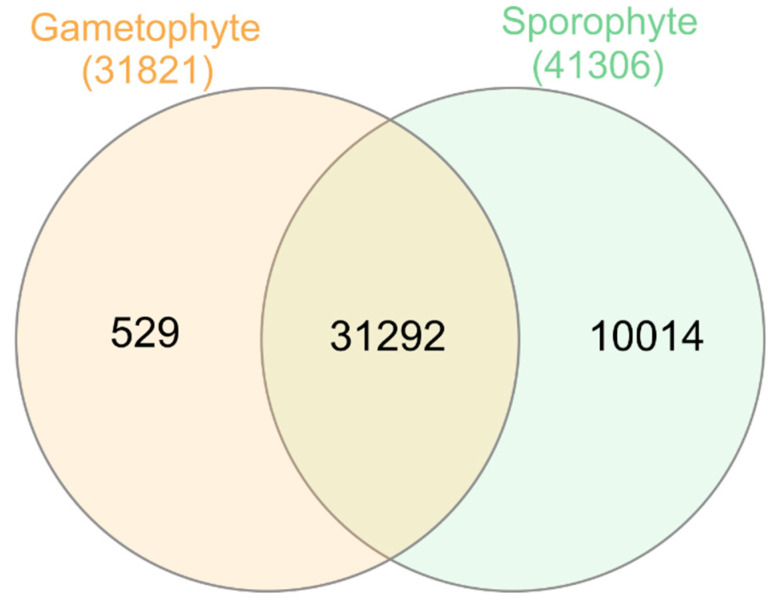
Overlapping and uniquely expressed transcripts per tissue. Colored numbers outside the Venn diagram show the total number of transcripts expressed in the gametophyte (**orange**) and the sporophyte (**green**).

The number of expressed transcripts was 31,821 in the gametophyte and 41,306 in the sporophyte, while 1,083 transcripts did not show mapped reads, according to our read count summary standards, in any tissue. There were 529 transcripts that were expressed in the gametophyte but not in the sporophyte (Supplemental Table S1), while 10,014 transcripts were expressed in the sporophyte but not in the gametophyte (Supplemental Table S2). The overlap between gametophyte and sporophyte-expressed transcripts was not significantly higher than expected by chance (*p*-value > 0.05, Figure 4). Among the 529 specific transcripts of the gametophyte, 258 were annotated, with 17% of them related to the stress response (defense and disease resistance, abiotic stress, etc.) and 7% being transcription factors, most of them involved in cell growth and differentiation, plant growth and development, as well as stress response. There were also two transcripts derived from transposable elements. Among the 10,014 specific transcripts of the sporophyte, only 3888 could be annotated. Of these, 1.5% of the transcripts were related to stress responses (defense and disease resistance, water deprivation conditions, abiotic stress, including salt and oxidative stress, both clearly related to drought and hydric stress, as well as iron and phosphate starvation). Besides, 3.4% (132 transcripts) were transcription factors, many of them involved in plant growth and development as well as stress responses. Eleven of the transcription factors expressed only in the sporophyte corresponded to different Knotted-like Homeobox genes, key for the distinctive gametophytic and sporophytic developmental programs [48,49,50,51,52], and one transcript corresponded to the Agamous-like MADS-box AGL16 protein that in flowering plants controls flower development [53]. There are also present two transcription factors of the GRAS family, of high importance as regulatory proteins in shoot and root development, stem cell homeostasis, light and hormone signaling, responses to biotic and abiotic stresses, and symbiosis with microorganisms [54]. In addition, 60 transcripts were involved in cell wall formation, including transcripts from genes involved in the synthesis of glucomannans, which constitute the type III primary cell wall in vascular plants and that are exclusively reported in some fern species [55,56]. Curiously, there were also 59 transcripts derived from transposable elements, most of them derived from non-LTR and LTR retrotransposons (43) but also from transposons (16). These elements, which represent 76% of the *V. speciosa* genome [57], seem to have high and differential activity between the two phases of the life cycle of the species.

Supplemental Tables S3 and S7 show the lists of the most expressed transcripts in the gametophyte and the sporophyte, respectively. The presence of transcription factors involved in development in flowering plants is remarkable among the 1000 most expressed transcripts in the gametophyte. Some of them are involved also in defense response and response to abiotic stress, such as water deprivation conditions. The existence of several transcripts for proteins that control the cell cycle, as well as those involved in the machinery of mRNA splicing, is also remarkable. There are also numerous transcripts related to stress responses (especially defense responses, water deprivation conditions, salt stress, oxidative stress, and osmotic stress) and to chloroplastidial functions. There were less transcription factors among the 1000 transcripts most expressed in the sporophyte, but this set included several transcripts related to cell wall formation, including transcripts from genes involved in the synthesis of glucomannans. The top 1000 most expressed transcripts in the sporophyte also showed transcripts for proteins that control de cell cycle and those involved in the machinery of mRNA splicing, besides many transcripts related to stress response (especially defense response, water deprivation conditions, salt stress, oxidative stress and osmotic stress) and to chloroplastidial functions.

This species is restricted to sheltered, very humid sites and is adapted to extreme low light environments [13,14,15]. Tables S1–S3 and S7 reflect these characteristics since an important fraction of the specific and/or most expressed transcripts are involved in plastid functions and responses to abiotic stresses. In addition, we can find differentiated patterns of gene expression that reflect the ecological, morphological, and physiological differences between the two phases of the life cycle of *V. speciosa*, such as transcripts from genes involved in cell growth, differentiation, and development, or a greater abundance of transcripts from genes involved in cell wall formation in the sporophyte.

**Figure 5:**
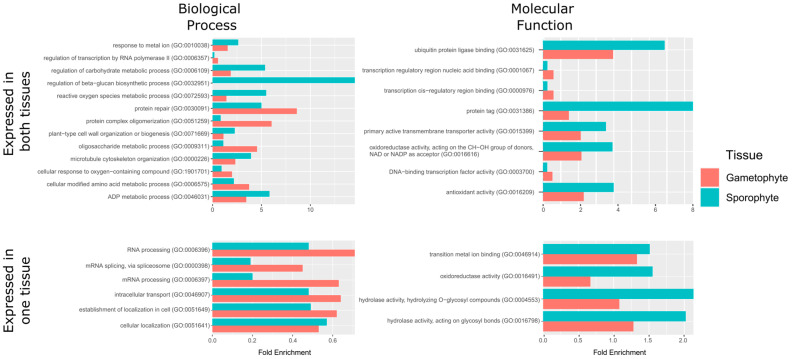
Enriched GO terms from the 1000 most expressed genes in the gametophyte and sporophyte (**top row**) and the transcripts expressed only in either the gametophyte or the sporophyte (**bottom row**). Each bar plot shows the fold enrichment of GO terms from biological process (**left column**) and molecular function (**right column**) ontologies. The X axis shows the fold enrichment of each term from either the gametophyte (red) or the sporophyte (blue) gene lists. The Y axis shows the GO term and its GO identifier. For the top 1000 transcript plots, only GO terms retained by the ReViGO analysis and showing the logarithm of the quotient between gametophyte and sporophyte fold enrichments greater than 0.5 are shown.

Analysis of enriched GO terms from the most expressed transcripts showed important differences between both the gametophyte and the sporophyte. The most expressed transcripts in the gametophyte (Table S3) showed 240 enriched GO terms from the three different ontologies: 141 from biological process, 27 from molecular function, and 72 from cellular component (Supplemental Tables S4–S6). The most expressed transcripts in the sporophyte (Table S7) showed 416 enriched GO terms: 230 from biological process, 73 from molecular function, and 113 from cellular component ontologies (Supplemental Tables S8–S10). The fold enrichment values from GO terms that were enriched either in the gametophyte or the sporophyte (whose redundancy has been filtered by ReViGO) are shown in Figure 5 and Tables S4–S6,S8–S10. As mentioned above, when comparing these enrichment values of the most expressed transcripts, we can observe differentiated patterns of gene expression between the two phases of the life cycle of *V. speciosa*. Comparing Tables S4 and S8, we can observe that there are abundant transcripts for proteins involved in metabolic processes, but they are differentiated between the two phases. For example, in the sporophyte, the cinnamic acid and the phenylpropanoid metabolic processes predominates, polysaccharide metabolism, glycine metabolism, and purine metabolism (see Tables S4 and S8). Remarkably, cinnamic acid and phenylpropanoids are central intermediates in the biosynthesis of a set of products, including lignols (precursors to lignin and lignocellulose) among others (flavonoids, isoflavonoids, coumarins, aurones, stilbenes, catechin, and phenylpropanoids). The sporophyte transcripts for proteins involved in metabolic processes related to lignin, cellulose, and glucan biosynthetic pathways and cell wall organization and biogenesis in general are over-represented in this list. Also noteworthy are the transcripts for proteins involved in ATP synthesis, in cytoskeleton organization, or response to stress. However, the latter are much more over-represented in the gametophyte (water, osmotic, heat, salt, ROS, oxidative stress, etc.). Particularly noteworthy are those involved in water control, as highlighted earlier. Both, the sporophyte and the gametophyte have over-representation of transcripts related to plastid assembly, functioning and repair as well as to developmental processes. We can conclude that enriched GO terms related to metabolism and growth are more abundant in the sporophyte, whereas those related to adaptation to extreme conditions and light uptake are more abundant in the gametophyte. Johnson et al. [13] and Makgomol and Sheffield [15] proposed that a very low metabolic rate and effective use of available light are characteristics that allow the gametophyte of *V. speciosa* to survive in extreme conditions. Our data also support that both phases of the life cycle of *V. speciosa* are adapted to a constant water supply.

In conclusion, this study complements the previously published transcriptome assembly from *V. speciosa* sporophyte [19], this time including gametophyte-specific transcripts. Despite its limitations (constrained mostly by the availability of threatened fern individuals and highly contaminated gametophyte samples), the results of this work provide further fern genomic resources and new insights on fern evolution and physiology. The results are even more valuable since the target species is simultaneously a non-model organism and an endangered species. It is also noteworthy that the *in silico* decontamination method that we apply here can be useful for any heavily contaminated tissue, which should help omics studies of samples whose nature makes them always associated (contaminated) with biological material from other organisms. With the sequencing resources in this and our previous [19] study, we offer a reference transcriptome for the species, unlocking the performance of population genomics and phylogenomics studies on *V. speciosa*. Being the reproductive success one of the possible causes of the endangered status of the species, the availability of the gene and gene expression data should allow comparative studies on the association between changes in gene sequences or expression and changes of the fitness of individuals and populations of the species. Of course, the gene transcripts that we provide here and in [19] can serve as supporting evidence for gene prediction in a future *V. speciosa* genome project.

## Figures and Tables

**Table 1:** RNA sequencing statistics. The total number of read pairs and the total number of sequenced nucleotides are shown for all sequenced tissues, before and after the quality check (QC).

	Tissue	Raw Data	After QC
Number of paired-end reads	Gametophyte	48.7 million	24.7 million
	Combined tissues	115 million	89.9 million
	Sporophyte	66.3 million	65.2 million
Number of bases	Gametophyte	4900 million	2500 million
	Combined tissues	10,800 million	9090 million
	Sporophyte	6700 million	6590 million

**Table 2:** BLAST analysis species representation. The percentage of plant, animal, fungi and protzoa species detected among the 50 most represented species is shown for the four assemblies. The last row shows the percentage of BLAST hits assigned to *A. thaliana* for the whole BLASTx analysis.

Percentage of BLAST Hits	Taxon	Raw Gametophyte	*In Silico* Decontaminated Gametophyte	Combined Tissues	Sporophyte
50 most represented species	Plant	30	60	60	68
	Animal	40	30	30	26
	Fungi	24	10	10	6
	Protozoa	6	0	0	0
All species	*A. thaliana*	30.29	55.88	55.82	70.00

**Table 3:** *De novo* transcriptome assembly statistics. The figures shown represent the total number of transcripts, percentage of guanine cytosine, N50, N70, N9, and Ex90N50 statistics (see text for details), number of transcripts with length equal or less to the Ex90N50, sizes of the smallest and largest contigs, number of contigs greater than 1000 and 10,000 base pairs long, median contig length, average contig length, and total number of assembled bases. The columns show the three different approaches (*in silico* decontaminated gametophyte, combined gametophyte and sporophyte, and sporophyte transcriptomes), before and after quality check and filtering steps. The acronym bp stands for base pairs.

	Gametophyte		Combined Tissues		Sporophyte	
	Before Filtering	After Filtering	Before Filtering	After Filtering	Before Filtering	After Filtering
Total transcripts	44,455	43,139	88,383	42,918	84,759	36,430
Percent GC	45.48	45.47	45.23	45.18	45.18	45.18
Contig N50 (bp)	2101	2102	2264	2243	1955	2085
Contig N70 (bp)	1509	1400	1632	1640	1332	1511
Contig N90 (bp)	786	659	807	855	479	729
Ex90N50 (bp)	2236	2235	2511	2615	2039	2299
Number transcripts corresponding to the Ex90 peak	11,445	11,519	13,665	13,743	14,645	21,543
Size of the smallest contig (bp)	201	201	194	196	201	201
Size of the largest contig (bp)	9541	9541	16715	16715	13,225	13,224
Number of contigs greater than 1 Kb long	25,786	16,605	50,272	25,454	35,801	20,532
Number of contigs greater than 10 Kb long	0	0	36	20	18	12
Median contig length (bp)	1223	1227	1234	1299	722	1197
Average contig (bp)	1462.28	1465.00	1504.37	1534.61	1144.86	1437.37
Total number of assembled bases	65,005,800	63,198,512	132,960,361	65,862,246	97,037,551	52,363,571

**Table 4:** Number of proteins from the UniProt database on which the *V. speciosa* transcripts align along a percentage of their length. The first column shows the length of the interval of the BLAST results length, in increments of ten. For each de novo assembly, *in silico* decontaminated gametophyte (Gametophyte) and combined *in silico* gametophyte and sporophyte (Combined tissues), we showed the “Number of proteins” within a BLAST result length interval, as well as the “Accumulated number of proteins”, for either the assembly “Before filtering” and “After filtering” contigs by expression and clustering by homology. Protein homology was assigned to each contig through a BLASTx analysis against the UniProt database.

	Gametophyte				Combined Tissues			
	Before Filtering		After Filtering		Before Filtering		After Filtering	
Percentage of Covered Length Intervals	Number of Proteins	Accumulated Number of Proteins	Number of Proteins	Accumulated Number of Proteins	Number of Proteins	Accumulated Number of Proteins	Number of Proteins	Accumulated Number of Proteins
91–100	3097	3097	3097	3097	3739	3739	3585	3585
81–90	1328	4425	1325	4422	1564	5303	1493	5078
71–80	938	5363	937	5359	1070	6373	997	6075
61–70	649	6012	646	6005	769	7142	690	6765
51–60	589	6601	587	6592	713	7855	619	7384
41–50	653	7254	648	7240	753	8608	635	8019
31–40	634	7888	623	7863	777	9385	623	8642
21–30	769	8657	762	8625	866	10,251	653	9295
11–20	849	9506	844	9469	1036	11,287	722	10,017
1–10	427	9933	426	9895	584	11,871	395	10,412

## Data Availability

The resulting database was deposited in FigShare, https://figshare.com/; https://doi.org/10.6084/m9.figshare.21502005, accessed on 4 November 2022.

## Supplementary Materials

The following supporting information can be downloaded at: https://www.mdpi.com/article/10.3390/genes14010166/s1. Figure S1: Contig coverage distribution per transcriptome; Table S1: Transcripts expressed only in the gametophyte; Table S2: Transcripts expressed only in the sporophyte; Table S3: Top 1000 most expressed transcripts in the gametophyte; Table S4: Biological process GO term enrichment analysis for the gametophyte; Table S5: Molecular function GO term enrichment analysis for the gametophyte; Table S6: Cellular component GO term enrichment analysis for the gametophyte; Table S7: Top 1000 most expressed transcripts in the sporophyte; Table S8: Biological process GO term enrichment analysis for the sporophyte; Table S9: Molecular function GO term enrichment analysis for the sporophyte; Table S10: Cellular component GO term enrichment analysis for the sporophyte.
