# Supplementary figures and images for: Comparison between the Gametophyte and the Sporophyte Transcriptomes of the Endangered Fern Vandenboschia speciosa
Source: Genes (Basel). 2023 Jan 7;14(1):166. doi: 10.3390/genes14010166 (PMC9859580; doi:10.3390/genes14010166)

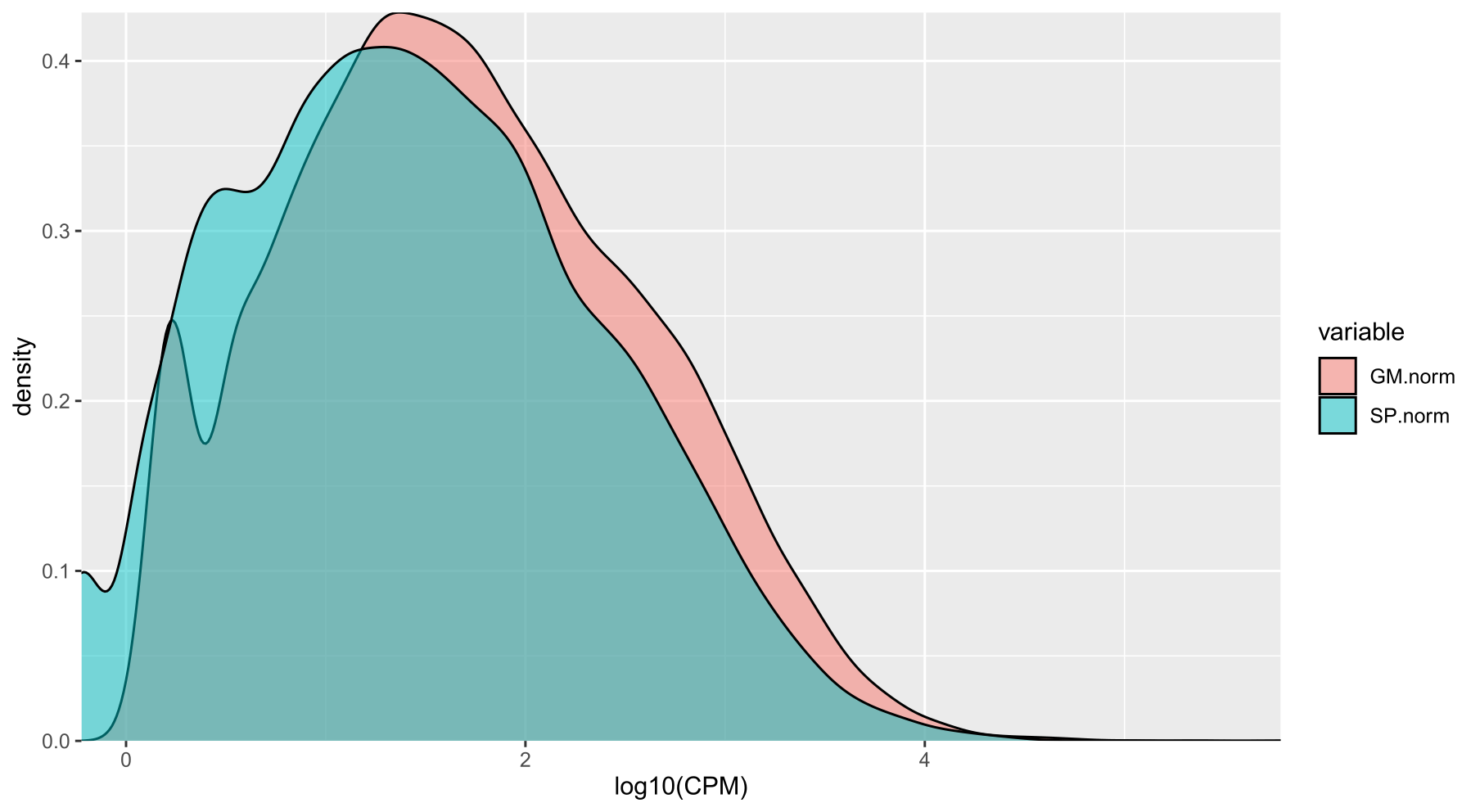

Supplement: Supplementary file 1 [file genes-14-00166-s001.zip › Supplementary_material/FigureS1.png]
